# Smallholder Farms and the Potential for Sustainable Intensification

**DOI:** 10.3389/fpls.2016.01720

**Published:** 2016-11-17

**Authors:** Leah M. Mungai, Sieglinde Snapp, Joseph P. Messina, Regis Chikowo, Alex Smith, Erin Anders, Robert B. Richardson, Guiying Li

**Affiliations:** ^1^Department of Geography, Environment, and Spatial Sciences, Michigan State University, East LansingMI, USA; ^2^Center for Global Change and Earth Observations, Michigan State University, East LansingMI, USA; ^3^Department of Plant, Soil and Microbial Sciences, Michigan State University, East LansingMI, USA; ^4^Department of Crop Science, University of ZimbabweHarare, Zimbabwe; ^5^Department of Community Sustainability, Michigan State University, East LansingMI, USA

**Keywords:** sustainable intensification, agriculture, Malawi, smallholder farmer, integrated management

## Abstract

The sustainable intensification of African agriculture is gaining momentum with the compelling need to increase food and agricultural production. In Southern Africa, smallholder farming systems are predominately maize-based and subject to erratic climatic conditions. Farmer crop and soil management decisions are influenced by a plethora of complex factors such as market access resource availability, social relations, environment, and various messages on sustainable farming practices. Such factors pose barriers to increasing sustainable intensification in Africa. This paper characterizes smallholder farming practices in Central Malawi, at Africa Research in Sustainable Intensification for the Next Generation (Africa RISING) project sites. We present findings from a survey of 324 farmers, located within four Africa RISING sites selected in a stratified random manner to represent (1) low agricultural potential (high evapotranspiration, variable rainfall), (2) medium agricultural potential (two sites), and (3) high agricultural potential (well-distributed rainfall). Soil fertility was low overall, and certain farming practices appeared to limit the sustainability of agricultural production. Nearly half of farmers did not value legume residues as a high nutrient value resource for soil amelioration, as legume residues were removed (17.9%) or burned (21.4%). Conversely, maize residues were rarely removed (4.5%) or burned (10.4%). We found that farmers do not allocate soil amendment resources to legume fields (zero instances of mineral fertilizer or manure application to legumes compared to 88 and 22% of maize systems, respectively). Policy makers in Malawi have led initiatives to intensify agricultural systems through subsidizing farmer access to mineral fertilizer as well as maize hybrid seed, and only rarely to improved legume seed. In this survey, farmers allocate mineral fertilizer to maize systems and not legume systems. There is urgent need to invest in education on sustainable reinvestment in natural resources through complementary practices, such as maximization of biological nitrogen fixation through improved legume agronomy and better organic resource and crop residue management. Recent efforts by Malawi agricultural services to promote doubled-up legumes as a sustainable intensification technology are encouraging, but benefits will not accrue unless equal attention is given to an extension campaign on management of organic resources such as crop residues.

## Introduction

Agricultural development in sub-Saharan Africa faces challenges from climate change, natural resource degradation, persistent food insecurity, and increasing intensification pressures from the millions of people whose livelihoods are rooted in smallholder farming. Godfray et al. (2010) argue that to address these challenges, more food needs to be produced in sustainable ways as compared to use of unsustainable practices that contribute to continuous loss of biodiversity and land overuse that causes land degradation (Vitousek et al., 1997).

Agricultural intensification practices that increase food productivity are often equivocal in terms of environmentally sustainability (Pretty, 2008; Petersen and Snapp, 2015). Sustainable Intensification (SI) of agriculture is an approach of agricultural production whereby desired outputs are increased without adversely affecting the environment or expanding the agricultural footprint (Giller et al., 2015). The important features of such an agricultural system include: producing more output per unit area; accruing natural, social, and human capital; and increasing the flow of environmental services (Godfray et al., 2010; Pretty et al., 2011; Giller et al., 2015). Yet, putting SI into practice is complicated by divergent understandings of goals, the sometimes challenging implementation of SI practices for farmers, temporal delays in positive returns or yield increases, and limited supportive policy frameworks for sustainable agriculture (Pretty, 2008; Petersen and Snapp, 2015).

Nevertheless, SI approaches have been considered to promote improved management of natural resources with attention to minimizing tradeoffs between productivity and profitability (Garnett et al., 2013; Kaczan et al., 2013; Pretty and Bharucha, 2014). Agricultural technologies that are often promoted as supporting pathways to SI include Conservation Agriculture, Integrated Soil Fertility Management (ISFM), and Climate Smart Agriculture (Place et al., 2003; Giller et al., 2015). Factors that limit the sustainability of agricultural development are not only global climate and market-economics related, but also related to any community’s access to education, health care, and infrastructure. Farmers might be unable to reach markets to access fertilizer and seeds or sell produce. Many face labor shortages, limited farm credit access, and poor governance (Sumberg, 2005; Pretty et al., 2011). These elements likely lead to constrained farmer decision making processes, as well as a disconnection between farmer knowledge and use of technologies (Sumberg, 2005; Tchale, 2009).

Rather than focus on specific practices like many of the above-mentioned approaches to SI, in this paper we focus on principles of SI: resource conservation, promotion of agrobiodiversity, building on local knowledge, and assisting farmers to incorporate modern innovations (Petersen and Snapp, 2015). We review the challenges and opportunities for SI of maize-based cropping systems on smallholder farms in Malawi. A survey of farmers elucidates current practices, and farmer perceptions that are relevant to many southern and east African smallholder maize-based systems.

### Background

Sustainability of Africa’s natural resources, conserving biodiversity, and enhancing current farming practices has not always been a priority of international agricultural research and development initiatives. Nonetheless, efforts are underway to develop African agriculture by promoting the revitalization of sustainable farming practices in partnership with smallholder farmers, extension advisors, and local communities (Snapp, 2004). These efforts seek to understand current farmer-agroecosystems, identify cultural constraints, and explore options for improving crop production (Giller et al., 2011). Additionally, there is a progressive shift toward agricultural research, technology accessibility, and implementation that involves the public and decision makers (Ochieng, 2007).

A practical tool for African agricultural development is through the use of “bottom-up” approaches where agricultural scientists co-learn with farmers and collaborate on developing options that are appropriate to local priorities, livelihoods, and practices (Altieri, 2002; Snapp S. et al., 2002). Most farmers have knowledge of diverse crops such as cassava, millet, and sorghum that contribute to the resilience of their farms to climatic variability (Rufino et al., 2013). Other practices that promote sustainable production include combinations of organic amendments with suitable fertilizers and the use of modern seed varieties (Pretty et al., 2011). Soil management techniques that improve water infiltration and storage are very important in rainfed systems for mitigating variable rainfall patterns across space and time (Krull et al., 2004; Thierfelder and Wall, 2009; Turmel et al., 2015). However, the use of organic inputs such as compost and incorporation of legume residues has been shown to vary widely in terms of the intensity of use by farmers (Valbuena et al., 2011).

In this paper we focus on the characterization of Malawian smallholder agriculture as a case study for regional agricultural production challenges and development opportunities. In the last two decades, Malawi maize production has seen wide variability driven by climate and policy related issues with a range of strategies promoted by the government and international debates (Dorward and Chirwa, 2011). Regular droughts threaten farmers’ livelihoods and food security thus in the late 1990’s, to alleviate drought risk, the Malawi government, with the assistance of international aid, implemented the use of a “starter pack” of affordable inputs of maize seeds and fertilizer to poor farmers (Levy, 2005, p. 274). Further, policies formed in the early 2000’s focused on inputs subsidies for fertilizers, and modern hybrids to improve production across the Malawi smallholder farmer sector (Dorward and Chirwa, 2011). There is considerable debate regarding implementation of the input subsidy program and also its subsequent impact with regards to improving access to inputs, and overall agricultural productivity. Survey findings suggest farmers are growing more modern maize varieties, although the impact on drought-resilience has been moderate and disappointment among some farmers has led to disadoption (Snapp and Fisher, 2015). Subsidy impact on fertilizer access appears to vary markedly from year to year and with farmer socio-economic status (Tchale, 2009; Whiteside, 2014).

Notwithstanding these challenges, Malawian farmers have experience with indigenous systems aimed at improving land and food quality, preserving soil moisture, and preventing soil erosion (Mulwafu, 2011). Farmers historically used shifting cultivation known as ‘makusa’ that improved soil fertility. Through this process, farmers gathered and burned tree branches and grasses, then mixed the ash with soil, and grew maize, cucumbers, and pumpkins. On the sloping hills of Shire highlands, the mound cultivation ‘matuto or katuto’ technique was used. These were flat mounds used for planting sweet potatoes and cassava, and intercrops of beans and groundnuts. In the plains, farmers cleared and tilled the ground ‘kulima pansi or chitipula’ before sowing maize, cowpeas, pigeon peas, (mphonda) edible gourds and sorghum (Mulwafu, 2011). Many of these practices have become challenging to practice given very limited use of fallows and diverse rotations given the small size of land holdings (due to rapidly growing population and government policies on land allocation, Jayne et al., 2014). Another traditional practice well suited to high labor availability is the production of compost, which could help meet urgent soil rehabilitation requirements. Some farmers used the compost heaping systems known as ‘Changu’ (turned and watered regularly) and ‘Chimato’ (covered with mud and static) to improve soil fertility (Nalivata, 2007).

At the foundation of sustainable practice is the production and management of crop residues. Intensification of crop production can lead to greater residue biomass, but this must be managed properly in order to build soil quality. There is a debate in the agronomic literature on whether crop residues should incorporated early – directly after crop harvest – as a means to enhance soil nitrogen stocks and biological processes, or left on the soil surface as a mulch to prevent erosion (TerAvest et al., 2015). The quality of the residues matters, as the decision tree developed by Palm et al. (2001) illustrates: low quality residues are well suited to erosion control, and medium to high quality residues (with a narrow C: N ratio, and biochemical constitutes that support rapid decomposition) are better suited to incorporation. Long-term benefits are derived from early incorporation of residues, but given that associated labor demands are high (as at harvest time residues tend to be high volume and the soil difficult to turn over), the deferred gains in soil organic matter pose challenges to farmer adoption (Vanlauwe et al., 2002). Mulch management can also have high labor requirements due to livestock control requirements and the transfer of biomass often recommended (Thierfelder et al., 2015); indeed, the soil cover practices associated with conservation agriculture have been critiqued as having limited relevance to smallholder farmer systems in Africa (Giller et al., 2009).

Different regions of Malawi employ specific crop residue management practices. Valbuena et al. (2015) reported that in the Mzimba area of northern Malawi farmers who do not own livestock tend to have more residue biomass available. In Southern Malawi, community norms are employed to control livestock year round (Rogé et al., 2016). Some studies have observed that the usage of crop residue depends on four related elements: farmers’ decisions, food production quantities, access to other biomass sources, and biomass requirements (de Leeuw, 1997; Erenstein, 2011).

### Africa RISING-Malawi Sites

The Africa Research in SI for the Next Generation (Africa RISING) program is funded by the United States Agency for International Development (USAID) as a part of the United States Government’s Feed the Future initiative. The program aims to improve food security, farmer livelihoods, and agroecological indicators of system health through the SI of key African farming systems, notably maize-based, rainfed production by use of action research. Africa RISING sites in Central Malawi were selected as representative of the widespread maize-mixed systems that encompass over 250 million hectares in sub-Saharan Africa (Blackie and Dixon, 2016).

The Africa RISING project identified entry points for SI of maize-based farming based on integrated nutrient management, judicious fertilizer use combined with enhanced legume presence, and improving utilization of legume products and residue management practices. In particular, pigeon pea was identified as a nitrogen-fixing leguminous crop producing protein-rich grain and substantial biomass that can be used for soil improvement as well as for multiple benefits, including forage and compost (Snapp et al., 2010). During the first year of the project, farmer practice was surveyed to assess current practice. Action research was initiated in the beginning of the growing season (November 2012), to introduce modern varieties of legumes and doubled-up legume technologies, including mixtures of pigeonpea and soybean as well as improved varieties of groundnut and cowpea. This key SI farming technique involves growing a two-legume intercrop in the first season, followed by intercropped pigeonpea and maize or sole maize in the second season (Snapp and Silim, 2002). Residue management of the doubled-up legume system is crucial to obtaining benefits for soil fertility as well as enhanced harvests (two crops) per land area (Bezner Kerr et al., 2007).

Simulation studies suggest that climatically risky sites may benefit from this doubled-up legume technology, although this requires extensive testing on-farm (Smith et al., 2016). Further, simulation research in Mozambique highlights the role that crop residues and management practices play as key regulators of nutrient retention and organic matter inputs to build soil carbon and nutrient pools (Rusinamhodzi et al., 2015). Thus, understanding how farmers practice integrated management, and in particular residue management, is essential information for assessing performance and SI potential across marginal and mesic sites.

## Materials and Methods

### Description of Sites

**FIGURE 1:**
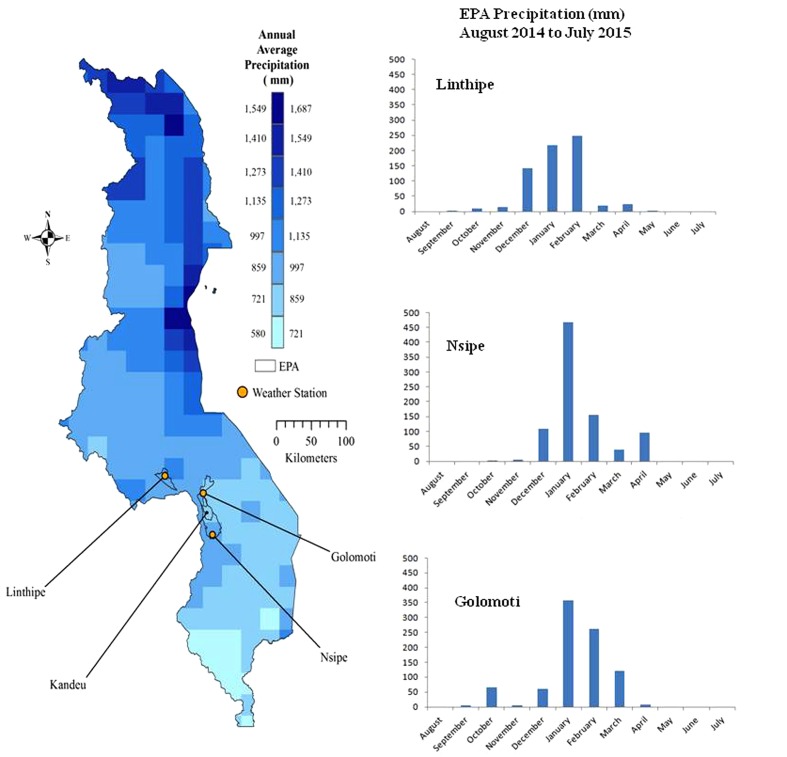
**Malawi Annual Average Precipitation (mm) based on Tropical Rainfall Measuring Mission (TRMM) rainfall data for 2001–2015; and monthly rainfall amounts of selected Extension Planning Areas (EPAs) from Weather Station datasets for August 2014 to July 2015 (Linthipe 667 mm, Golomoti 884 mm, and Nsipe 875 mm)**.

The study sites were chosen using a stratified random approach, where four sites were chosen along a gradient of agroecological zones from low to high production potential across central Malawi (Dedza and Ntcheu districts). The locations were randomly chosen extension sections (with several villages located within each section), within Golomoti and Linthipe Extension Planning Areas (EPA) in Dedza, and Kandeu and Nsipe EPAs in Ntcheu. The four EPAs vary in geophysical features as Malawi’s land surface straddles the North West-to-South East, low-to-high elevation parts of the African rift valley^1^ (Brown and Young, 1965). The varying geographical gradient, and climatic conditions play a role in influencing productivity. As shown in **Table 1**, Golomoti is a low agricultural potential site located at low elevation, with high evapotranspiration and variable rainfall, Kandeu and Nsipe are medium agricultural potential, located on medium elevation, with medium rainfall and Linthipe is a high agricultural potential, high elevation site, and well-distributed rainfall (Tamene et al., 2015; Smith et al., 2016). Malawi has a unimodal rainy season occurring from November to April, and a dry season from May to October (Jury and Mwafulirwa, 2002). **Figure 1** shows the long-term annual average rainfall for Malawi from 2001 to 2015 using Tropical Rainfall Measuring Mission (TRMM) dataset, and monthly weather station data graphs from August 2014 to July 2015 for the three selected EPAs. The rainfall pattern shown here illustrates the regional spatial and temporal variability (Ngongondo et al., 2011; Kumbuyo et al., 2014).

**Table 1:** Environmental and physical farming system characteristics of four sites in Central Malawi based on spatial data from various sources^1-6^ and surveys conducted in July of 2013.

	Golomoti	Kandeu	Nsipe	Linthipe
	
Productivity potential	Low	Medium	Medium	High
**Physical characteristics**				
Latitude/Longitude^2^	14.32°S/34.66°E	14.65°S/34.68°E	14.80°S/34.72°E	14.26°S/34.10°E
Elevation local point (meters above sea level)	555	904	868	1238
Elevation^3^ (meters above sea level)	504	877	967	1248
TRMM Annual^4^ Average rainfall (mm)	895	866	866	953
Local rainfall (mm)	884	–	875	667
Evapotranspiration^5^ (mm)	960	619	607	595
EPA mode soil^6^ suitability	Moderately suitable	Marginally suitable	Marginally suitable	Moderately suitable
Primary income sources	Crop sales; small business	Crop sales; Horticulture	Crop sales; small business	Crop sales; farmer laborer (Ganyu)
Distance from small market (km)	1	2	9	5
Distance from large market (town) (km)	40	35	20	40


*^1^http://www.malawiproject.org/about-malawi/geography/ accessed 11 June 2016. ^2^Latitude and Longitude values derived from the Extension Planning Areas (EPA) polygon (source: GCS 1984, UTM zone 36S). Used ArcGIS to dissolve extra polygons within the same EPA, converted polygon features to points, and created a centroid point, used calculate geometry option to get each centroid point in degree (units). ^3^Digital Elevation Model, Shuttle Radar Topographic Mission (SRTM) 90 m: spatially joined with EPA polygons, calculate zonal statistics for each EPA. ^4^Tropical Rainfall Measuring Mission (TRMM) Annual Average Rainfall: spatially joined TRMM and EPA polygons, calculated EPA average rainfall (mm). ^5^Global long-term (1983–2006) daily Evapotranspiration (1°): spatially joined with EPA polygons, calculate zonal statistics for each EPA. ^6^Source: Africa Soil Information Service (AfSIS) soil dataset, calculated mode-zonal statistics per EPA using spatial analyst in ArcGIS.*

**FIGURE 2:**
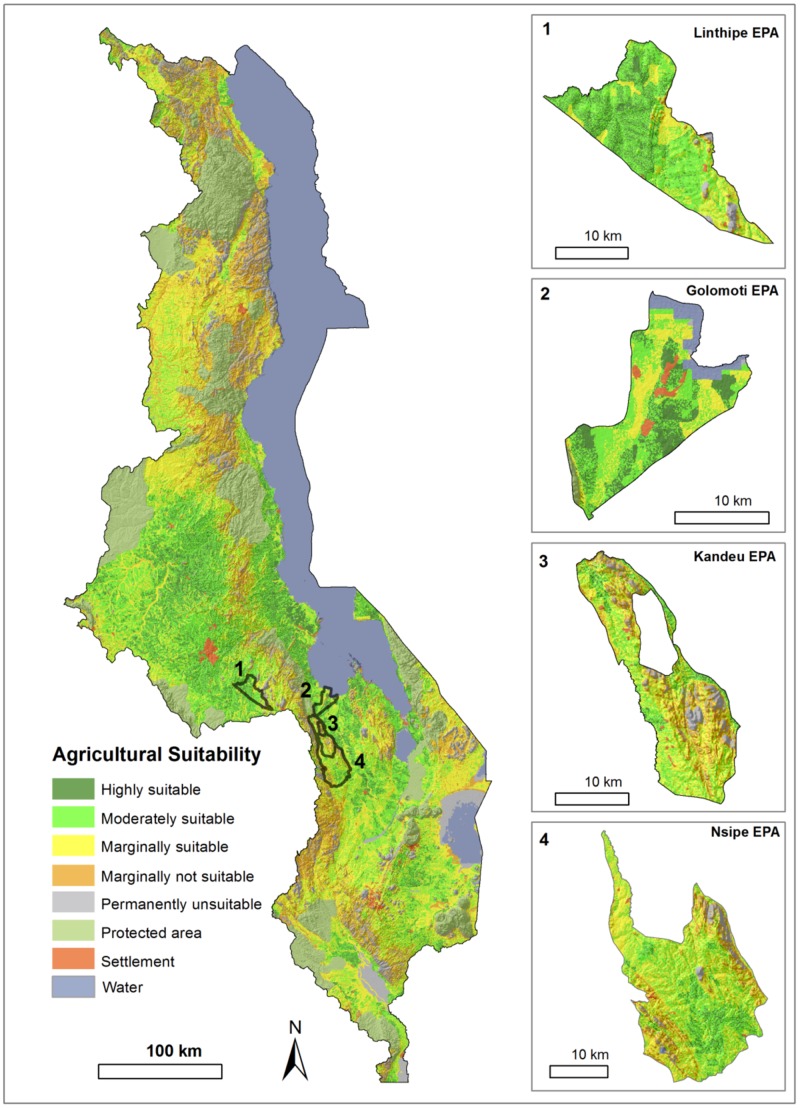
**Malawi agricultural land suitability^2^ Map that highlights the four EPA regions**.

Soils at Golomoti tend to be coarse and a mix of eutric cambisols and eutric fluvisols, the Kandeu and Nsipe sites are dominated by mixed chromic luvisols and orthic ferralsols, while Linthipe are primarily ferric luvisols (Lowole, 1983). Soils in these locations were characterized further as seen in **Figure 2**. The Malawi agricultural land suitability was assessed based on eight terrain and soil factors including soil erosion risk, soil organic carbon, soil texture, soil depth, soil exchange capacity, soil drainage, and soil pH derived from the Africa Soil Information

Service (AFSIS) soil dataset, and terrain slope from SRTM DEM (Shuttle Radar Topographic Mission 90 m). We combine several empirical models such Square Root, Storie, Rabia, Weighted average, Geometric Mean. All variables were quantitatively rated, and grouped into eight soil suitability classes (Storie, 1978; Rabia and Terribile, 2013; Pourkhabbaz et al., 2014; Li et al., submitted).

### Survey Method

A semi-quantitative interview instrument was used to survey 324 farm families over a 10-week period from late-May to late-July, 2013. This survey dataset included intervention, local control, and distant control households from 22 village clusters in two districts including Dedza (Linthipe and Golomoti) and in Ntcheu (Kandeu and Nsipe). Note that Mtakataka is adjacent and highly similar to Golomoti and was combined with Golomoti for this study. Preliminary statistical analyses showed no difference in farming practices between intervention farmers and control farmers, which is not surprising as this was a survey conducted as part of a baseline characterization exercise.

The sample consisted farmers who participate in an Africa RISING research project that was started in 2012, and near control and distant control farmers chosen using a “Y-sampling frame.”

The survey instrument was approved through the MSU IRB human subjects protocol, and translated into local languages with information provided as to the voluntary nature of the survey, and every effort was carried out to maintain confidentiality. Enumerators were trained over a 1-week period, and supervised in the field by graduate students, and the data collection process included close attention to data entry and data quality control, as described in an earlier report on the gendered-aspects of farmer experimentation reported in this survey and in a complementary qualitative research project (Hockett and Richardson, 2016).

The total participants comprised 97 males and 227 females. Participants from Dedza district were a total of 163 (about 2.61% of the population of Dedza) and 161 participants were from Ntcheu district (3.41% of the population of Ntcheu). The survey involved 71% male-headed households and 29% female-headed households, which is a typical distribution of household characteristics in Central Malawi (World Bank, 2016).

The survey topic addressed socio-economic characteristics such as household size, dependency ratio (calculated as the number of individuals who are either younger than 15, or older than 65, relative to adult members of the family who contribute fully to agricultural labor ^∗^ 100, Hockett and Richardson, 2016). The survey also asked questions about farm management, including detailed information on practices in the 2012/2013 season on a field by field basis, e.g., crops grown, intercrops with maize systems and other cropping systems, soil fertility practices, and types of animals owned. This allowed characterization of baseline farming activities and social situation. Note that we do not report here on the small experimentation plots some of the Africa RISING intervention farmers started carrying out in 2012, as information on these plots was excluded from the analyses. We were interested in the common farm practices across the entire Central Malawi area where this work is being carried out including among farmers not interacting with the Africa RISING projects (indicated here as control farmers). All crops grown per plot were documented in the survey. We report here on the most important cropping systems as characterized by the first two reported species. For example, the maize + pigeonpea intercrop included a few instances of maize + pigeonpea + cowpea, and maize + groundnut included a few instances of maize + groundnut + common bean.

Soil fertility measures including compost and manure application, residue management and fertilizer use were asked about on a field by field basis, which allowed evaluation of use by cropping system as well as by farm household. Field size was reported for a subset of fields, 70% of the 657 fields reported in the survey, and those fields were used to calculate application rate of fertilizer. Compost and manure use was asked about separately in the survey, but in general animal manure was a small proportion of amendments applied and included aged manure dug out of confined livestock areas which is similar to compost. Based on the literature, both compost and manure are very low in nutrient status on smallholder farms (about 1% or less N; Vanlauwe et al., 2002); thus, we combined manure and reported it within the compost category.

### Natural Resource Management Context

To explore the sustainability in relationship to a variable natural resource management context and farmer practices we present data from the survey and predict crop yield and soil organic matter trends over time at the three sites where we have conducted crop and soil modeling simulations Golomoti, Kandeu, and Linthipe (Smith et al., 2016). The simulations are based on the analysis by Smith et al. (2016) that used the Agricultural Production Systems Simulator (APSIM) crop and soil simulation model. APSIM has been used widely over a decade in the sub-Sahara Africa region and has been validated using crop yield data from sole, intercrop, and rotational systems of maize-legume crops (Keating et al., 2003; Whitbread et al., 2010).

Here the simulation analysis was carried out using a subset of this survey dataset collected in 2012–2013 season and 2013–2014 growing seasons for maize-legume rotation low potential-Golomoti, medium potential-Kandeu, and high potential-Linthipe. Also, the APSIM model used ancillary datasets including soils and weather data, and parameters such as plant spacing, crop varieties, and crop residue removal practices for 26 growing seasons of a maize/legume rotation scenario nominally run from November 1, 1979 through June 30, 2005 (Smith et al., 2016).

## Results

### Socio-Economic Characteristics

Generally, smallholder farmers at the four sites have very limited resources. This is typical of the smallholder sector in Southern Africa. Household size comprised 5.1 persons with a dependency ratio of 104–112. An average of 2.8 persons from each household contributed labor to agricultural production (**Table 2**). The majority of farm households (88.9%) held two fields or fewer and cultivated an average of 0.85 hectares; food produced by the average household was reported to last 8.22 months. Farmers generally reported the sales of their agricultural produce from their rainfed fields as their key income source, with no secondary source of income. At Linthipe there was greater reliance on other income sources, compared to the other sites (**Table 2**). Several farmers own small animals, but only a few own cattle, and there was no reported use of animal traction. The low levels of livestock limit the use of animal manure in the fields. Farmland cultivation is labor intensive as hoes are used to break up the soil in preparation for sowing and to build planting ridges, as well as for manual weeding.

**Table 2:** Social characteristics for farmers in the four sites in Central Malawi based on surveys (*n* = 324 except where otherwise noted) conducted in July of 2013.

	Golomoti	Kandeu	Nsipe	Linthipe
**Extension Contact %**				
None	46	53	39	41
Received advice on one agricultural topic	14	11	20	12
Received advice on two agricultural topics	15	12	7	13
Received advice on three agricultural topics	25	24	34	34
**Household (HH)**				
Male HH head: n (%)	60 (75%)	56 (69%)	58 (73%)	57 (69%)
Female HH head: n (%)	20 (25%)	25 (31%)	22 (27%)	26 (31%)
Average HH size (persons)	5.1	5.2	5.1	5.2
Dependency ratio^1^	108	104	108	112
Avg. farm size (ha) (*n* = 600)	0.83	0.89	0.97	0.71
Avg. # of fields	1.89	2.38	2.4	2.24
Avg. # laborers available (from within HH)	2.61	2.81	3	2.76
Avg. # months food supply	7.16	7.83	9.65	8.24
**Major crops**	Maize, cotton, groundnut	Maize, tobacco	Maize, tobacco, groundnut	Maize, tobacco, groundnut
**Unique crops**	Cowpea	Groundnut, soybean	Soybean, sweet potato	Soybean, common beans
**Livestock ownership (%)**				
Cattle %	3.8	13.6	2.5	8.4
Goats %	46.3	37	50	45.8
Pigs %	17.5	14.8	30	19.3
Poultry %	62.5	80.2	81.3	72.3
**Average population per sq. km**^2^	75	150	75	150


*^1^Dependency Ratio as the number of age population not in the labor force divided by working – age population (World Bank, 2016). ^2^Source: Benson et al. (2002). Malawi Atlas of Social Statistics. Used population area averages for the traditional authorities’ map based on our EPA centroid.*

Overall, 45% of farmers reported having had no contact with extension agents during the previous seasons for agricultural information on topics such as crop/variety guidance, land preparation techniques, and fertility measures (**Table 2**). Of the 55% that received extension advice, about 20% received advice on one or two agricultural topics and the remainder reported contact with extension agents that addressed three or more agricultural areas. Contact with the extension educators was a higher percentage for Africa RISING participants than for control farmers, which was not surprising as this was a baseline study.

### Agricultural Potential

The agricultural land potential was categorized based on soil and environmental factors, with land being classified as follows: highly suitable (8.2%), moderately suitable (24.1%), marginally suitable (28.0%), and unsuitable (39.7%) of the total land area (**Figure 2**). The four EPA sites in our study were ranked based on the majority of pixels’ classification in each site: on this basis, Golomoti and Linthipe land was generally classified as moderately suitable, while Kandeu and Nsipe was classified as marginally suitable (**Figure 2**). The soil organic matter change over 25 years based on APSIM simulation of the most common cropping systems (continuous maize, maize-groundnut rotation and maize-pigeon pea intercrop), and range of maize response to N fertilizer for three low to high potential sites, Golomoti, Kandeu, and Linthipe (**Table 3**). This exercise highlights that soil organic matter has the potential to decline the most in Linthipe, the high potential site, particularly relative to Kandeu, a medium potential site. The model simulated response of maize to N fertilizer varied markedly over the three sites (13–116 kg grain/kg N/ha), but was much higher in maize response to fertilizer was also influenced by the cropping system, as maize systems with legumes present compared to continuous maize (**Table 3**), with implications of agricultural sustainability.

**Table 3:** Soil organic C status measured in 2014 and simulated change over 25 years using Agricultural Production Systems Simulator (APSIM), calibrated for three EPA sites in Central Malawi, and range of simulated maize nitrogen efficiency for continuous maize, maize-groundnut rotation, and maize-pigeonpea intercrop over 25 years at low and high potential sites (Adapted from Smith et al., 2016).

Measures	Cropping systems	Golomoti	Kandeu	Linthipe
Soil organic C change^∗^	Initial SOC	0.85	1.05	2.33
	Maize	-0.136	-0.1	-0.88
	Maize and groundnut	-0.036	0.076	0.72
	Maize and pigeonpea	0.112	0.456	0.088
Maize nitrogen efficiency (kg maize grain/kg N fertilizer per ha)	Maize	30.1–68.9	43.0–80.0	18.7–69.8
	Maize and groundnut	32.4–105.0	29.0–110.0	34.8–116
	Maize and pigeonpea	20.9–70.6	13.7–66.2	39.1–73.6


*^∗^% soil organic C (SOC) change over 25 years.*

### Cropping Systems

For the 2012–2013 season, farmers reported growing a variety of crops seen in (**Table 4**); 59% of farmers reported growing a maize hybrid variety and 75% reported growing a local variety, as might be expected as maize is the staple food of the region and grown for household consumption as well as sale. For maize hybrid varieties, Golomoti and Linthipe farmers grew about 60%, compared to Nsipe farmers who grew 70% and Nsipe was lower at 46%. More local maize varieties were grown in medium potential areas of Kandeu and Nsipe (81 and 86%), and relatively fewer in Golomoti (69%) and Linthipe (64%). Farmers grew legumes primarily for consumption and were either grown as a sole crop or intercropped with maize. Groundnut was grown by more than 50% of the farmers Nsipe, Kandeu, and Linthipe and grown significantly less frequently in Golomoti (25%). Common bean was grown by a majority of farmers (83%) in Linthipe in contrast with nearly 30% of farmers in Kandeu and Nsipe.

**Table 4:** Crop species grown in 2012–2013 growing season by farmers at four sites in Central Malawi, number of farmers reporting each crop shown, and percentage of farmers per site presented in parentheses.

Crops	Golomoti (*n* = 80)	Kandeu (*n* = 81)	Nsipe (*n* = 80)	Linthipe (*n* = 83)	Total (*n* = 324)
Local maize^∗^	55 (69%)	66 (81%)	69 (86%)	53 (64%)	243 (75%)
Hybrid maize	49 (61%)	37 (46%)	56 (70%)	49 (59%)	191 (59%)
Tobacco	1 (1%)	10 (12%)	9 (11%)	6 (7%)	26 (8%)
Cotton	24 (30%)	2 (2%)	0	1 (1%)	27 (8%)
Pigeonpea	11 (14%)	4 (5%)	17 (21%)	7 (8%)	39 (12%)
Groundnut	20 (25%)	46 (57%)	46 (58%)	42 (51%)	154 (48%)
Soybean	12 (15%)	24 (30%)	17 (21%)	31 (37%)	84 (26%)
Common bean	0	25 (31%)	22 (28%)	69 (83%)	116 (36%)
Cowpea	50 (63%)	12 (15%)	16 (20%)	6 (7%)	84 (26%)
Bambara nut	0	0	4 (5%)	0	4 (1%)
Sorghum	0	0	4 (5%)	0	4 (1%)
Cassava	0	2 (2%)	7 (9%)	0	9 (3%)
Sweet potato	2 (3%)	2 (2%)	4 (5%)	5 (6%)	13 (4%)
Irish potato	0	0	0	1 (1%)	1 (0.3%)
Cocoyam	0	1 (1%)	0	0	1 (0.3%)
Millet	0	33 (41%)	25 (31%)	0	58 (18%)
Rice	2 (3%)	0	0	0	2 (0.6%)
Pumpkin	3 (4%)	10 (12%)	28 (35%)	3 (4%)	44 (14%)
Tomato	0	0	1 (1%)	0	1 (0.3%)
Cucumber	1 (1%)	0	2 (3%)	0	3 (0.9%)


*^∗^Total maize grown exceeds sample size as some farmers grew both local and hybrid maize. Based on survey (*n* = 324) conducted July of 2013 in Central Malawi.*

Golomoti farmers did not grow common bean but rather 63% of farmers in Golomoti reported growing cowpea while farmers in the other three EPAs reported growing it infrequently (20% and less). Soybean was grown less commonly as reported by 26% of all farmers in all locations. Pigeon pea crop was infrequently grown in Nsipe (21%), Golomoti (14%), Linthipe (8%), and Kandeu (5%). Tobacco was grown by a few farmers in Kandeu (12%), Nsipe (11%), Linthipe (7%), and Golomoti (1%). Cotton was grown mainly in Golomoti as a cash crop, but infrequently (5%) in both Linthipe and Kandeu, and Nsipe farmers did not report growing it at all.

There were a number of farmers that reported growing other crops, such as millet (41%) in Kandeu, (31%) in Nsipe. Sweet potato was grown by only 3% of farmers in Golomoti, (2%) in Kandeu, (5%) in Nsipe, and 6% of farmers in Linthipe. One household in Nsipe grew tomatoes. Cocoyam was grown by one household in Kandeu, cucumber was grown by three households, Nsipe (2), and Golomoti (1). Rice was grown by two households in Golomoti. These alternative crops were locally important as intercrops with maize in specific communities, such as finger millet grown as an intercrop with maize by 41% of households in Kandeu and 26% of households in Nsipe; presumably for local use in beer brewing. Pumpkin and cucumber were grown as intercrops in 4% of fields in Linthipe, 5% in Golomoti, 12% Kandeu, and in 34% in Nsipe, but were not included as separate cropping systems.

The low potential site, Golomoti, exhibited a few unique farming characteristics, as about 50% of farmers reported growing cowpea with maize as a relay intercrop, where cowpea is planted before the maize crop is harvested, as compared to about 10% of farmers at Kandeu and Nsipe, and fewer farmers in Linthipe (2%). The cash crop cotton was also grown as a sole crop by a substantial number of Golomoti farmers, 25%, and grown by fewer farmers in Kandeu (2%) and Linthipe (1%). Pigeon pea was grown as part of a maize intercrop by about 13% of farmers in Golomoti, and 11% in Nsipe, and at lower levels in Linthipe and Kandeu (2 and 4%, respectively). A surprising result was that some farmers reported growing groundnut as an intercrop with another grain legume, particularly in Linthipe where farmers reported sowing groundnut and soybean (8%), and 1% in Nsipe, farmers reported growing groundnut and common bean (5%) in Nsipe, and 4% in Linthipe. Groundnut and cowpea was grown at about 2% in Linthipe, and 1% of farmers in Nsipe and Golomoti, respectively. Overall, legume crop area was markedly higher in Linthipe relative to the other sites (**Table 5**). This was due primarily to the popularity of a maize-bean intercrop at this mesic agricultural site present in about 80% of the maize area (**Table 4**).

**Table 5:** Farming system combinations grown at four sites in Central Malawi, percentage of farmers using the combination per site.

Crops	Golomoti (%)	Kandeu (%)	Nsipe (%)	Linthipe (%)
Maize (sole)	53.8	67.9	65	18.1
Maize + pigeonpea	12.6	2.4	11.3	3.6
Maize + groundnut	6.3	18.5	12.5	3.6
Maize + soybean	7.6	24.7	12.6	8.4
Maize + common bean	1.3	23.5	26.3	82.3
Maize + cowpea^∗^	50	11.1	15	2.4
Groundnut + soybean	0	1.2	0	8.4
Groundnut + common bean	0	0	5	3.6
Groundnut + cowpea	1.3	0	1.3	2.4
Pigeonpea (sole)	1.3	1.2	7.5	0
Groundnut (sole)	12.5	35.8	33.8	34.9
Tobacco (sole)	1.3	11.1	11.3	7.2
Cotton (sole)	25	2.5	0	1.2
Sweet potato (sole)	2.5	2.5	2.5	6
Soybean (sole)	1.3	6.2	7.5	20.5


*^∗^Cowpea grown as a relay crop with maize. Based on survey (*n* = 324) conducted July of 2013 in Central Malawi.*

**FIGURE 3:**
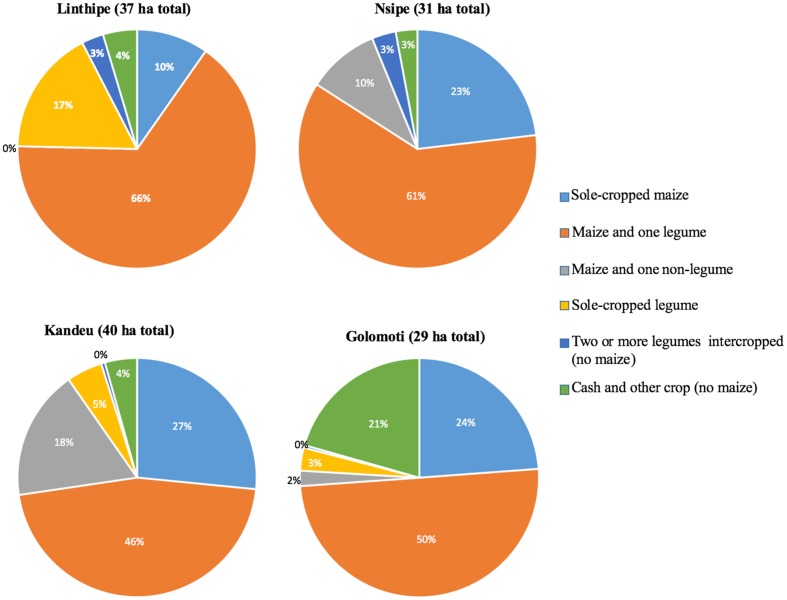
**Proportion of area devoted to each crop is shown in this figure for low (Golomoti), medium (Nsipe and Kandeu) and high (Linthipe) potential sites in Central Malawi based on project survey conducted July of 2013, fields *n* = 600**.

Overall, we see that the patterns of cultivation are more pronounced when presented on an area basis rather than a field basis (**Figure 3**). The area devoted to maize is substantially larger compared to the other crops grown four EPAs have diverse cultivation by area of cereals and legumes and also the arable land varies across the EPAs. Linthipe farmers reported 37 ha total area, and grew a maize-legume crop on over 60% of their fields, a sole legume also occupied 17% of the fields followed by other crops. Nsipe had 31 hectares reported, where farmers cultivated 61% of their fields with a maize-legume, followed by 23% of sole maize crop. Kandeu reported 40 hectares of land, with sole maize (46%) while 27% of land with atleast two-legumes, and 18% land had a maize and other cereal crop. Golomoti reported on 29% hectares, of which 50% was in a maize-legume intercrop, 24% sole maize, and a high allocation to cash and other crops, namely cotton (21%). The other three EPAs had less than 5% in cash and other crops.

### Fertilizer and Compost Manure Practices

Farmers were asked to report amendments applied in 2012–2013 on a field-by-field basis, the amounts and type of inorganic fertilizers, as well as compost and manure. Over 80% of surveyed farmers reported applying mineral fertilizer (average nutrient rate applied to fertilized fields was 65 kg N/ha; 21 Phosphate-P kg/ha) in one or more of their fields. The standard deviations for the fertilizer N and P applied suggests high variability in application dose, as does the range of fertilizer use shown across the four EPAs (**Table 6**). The average rate applied is close to the Malawi government recommended rate for N applied to hybrid maize, which is 69 kg N/ha. Surprisingly, the rate applied to fertilized fields of P fertilizer was higher than the recommended rate of 9 kg Phosphate-P kg/ha. However, we note that a recent convening of Malawi agricultural experts report indicated that maize fertilizer recommendations are shifting, toward the recommendation from the early 1990’s which was a country-wide blanket recommendation of 92 kg N/ha and 45 kg Phosphate-P kg/ha, plus 4 kg S/ha (Mutegi et al., 2015). The proportion of farmers who applied fertilizer was consistently high across all sites, from 76% at the marginal Golomoti site to 93% at the Nsipe site. This likely reflects the apparent effectiveness of Malawi government policies that have emphasized subsidization of fertilizer to improve farmer access (Dorward and Chirwa, 2011). The lowest amounts of nutrients were applied in Golomoti, at 52 kg N/ha and 14 Phosphate-P kg/ha. This is to be expected, as rainfall variability and marginal soils are often associated with moderate doses of fertilizer as a risk mitigation strategy (Kurwakumire et al., 2014). The APSIM simulated response of maize to N fertilizer varied markedly over the three sites (13–116 kg grain/kg N/ha), but was much higher in maize systems with legumes present compared to continuous maize (**Table 3**).

**Table 6:** Inorganic fertilizer use, compost and residue management practices at four sites in Central Malawi, percentage of farmers using the combination per site.

	Golomoti (*n* = 54)	Kandeu (*n* = 58)	Nsipe (*n* = 55)	Linthipe (*n* = 53)	Total (*N* = 220)
**Fertilizer management**					
% applying mineral fertilizer	75.9	82.8	92.7	79.2	82.7
N rate where applied (kg/ha)^∗^	52	65	70	70	65
N rate standard deviation	43	48	46	38	45
N rate range	0.07–21.6	1.42–37.2	1.06–42.6	2.27–21.3	
P rate where applied (kg/ha)^∗^	14	24	20	22	21
P rate standard deviation	10	21	14	12	15
P rate range	0.06–6.4	0.32–12.9	0.32–7.8	0.86–6.5	
% applying manure/compost	25.9	22.4	30.9	43.4	30.5
**Residue Management**					
% incorporated residues early	29.6	58.6	18.2	39.6	36.8
% incorporated residues late	29.6	34.5	72.7	28.3	41.4
% burned residues	37	13.8	16.4	37.7	25.9
% removed residues	5.6	15.5	0	22.6	10.9


*^∗^Fertilizer rate is in relationship to fertilized fields. This is based on survey subset of farmers with detailed fertility management and area questions (*n* = 220) conducted July of 2013 in Central Malawi.*

Thirty percent of all farmers reported applying organic amendments (compost or animal manure). Linthipe farmers reported the highest rate of organic amendment use (43%). At the other locations 31% or fewer farmers reported applying an organic amendment to one or more of their fields (**Table 6**).

**FIGURE 4:**
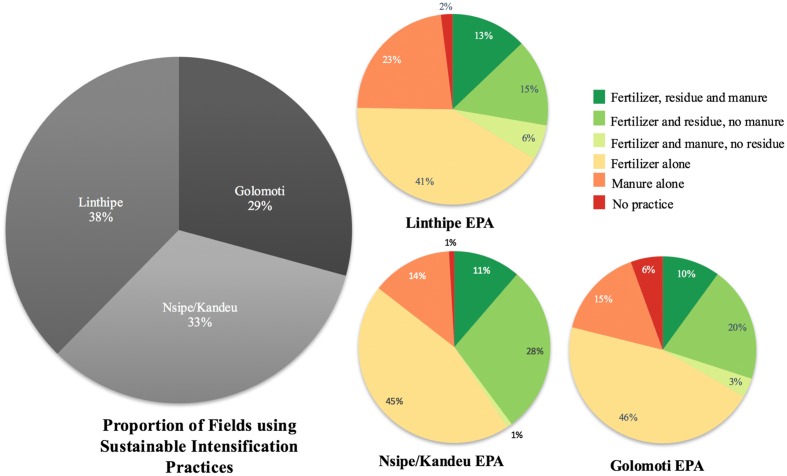
**Proportion of area devoted to field management practices is shown in this figure for low (Golomoti), medium (Nsipe and Kandeu), and high (Linthipe) potential sites in Central Malawi based on project survey conducted July of 2013, fields *n* = 600.** The overall pie chart shows the proportion of area devoted to combined practices that represent sustainable intensification, those in shades of green (fertilizer combined with an organic amendment such as residue incorporation or manure).

Mineral fertilizers were overwhelmingly applied to systems that included maize or a cash crops; 88–89% to sole maize and maize intercrop systems, and 48% to a cash crop (**Table 7**). There were zero instances of mineral fertilizer application in sole legume crops. Likewise, organic soil amendments were not applied to sole legumes. Sole maize and maize non-legume intercrops were amended with compost or manure by about 21% of farmers, whereas maize-legume intercrops were amended by 35% of farmers (**Table 7**). In terms of use of SI practices, few farmers are using all organic nutrient sources (10–12%), but many farmers are combining residue incorporation, or compost application, with fertilizer use, about 40–50% of fields (**Figure 4**). Fertilizer use alone was common, with about 42–47% of farmer fields using no residue incorporation or compost to ensure sustainable management of fertilizer. There was no clear pattern of environmental context influencing SI practice, as practices were approximately evenly distributed across sites (**Figure 4**) and no significant differences between the control and participant farmers on farm practices, and thus the outputs were based on EPA sites.

**Table 7:** Inorganic fertilizer use, compost, and residue management practices by cropping systems, percentage by cropping system.

	MzSol (*n* = 67)	MzLeg (*n* = 137)	MzOth (*n* = 25)	LegSol (*n* = 28)	LegLeg (*n* = 7)	Cash and other (*n* = 33)
Applying mineral fertilizer	88.1	86.9	88.0	0.0	0.0	48.0
N rate where applied (kg/ha)	62	62	76	N/A	N/A	75
N rate Standard Deviation	53	40	59	N/A	N/A	57
N rate range	11–341	6–227	17–227	N/A	N/A	0.60–170.5
P rate where applied (kg/ha)	20	19	21	N/A	N/A	28
P rate Standard deviation	17	14	16	N/A	N/A	19
P rate range	1.3–103.1	1.3–69.1	5.1–51.9	N/A	N/A	0.51–51.9
% applying manure/compost	22.4	35.0	20.0	0.0	0.0	9
**Residue Management by Cropping System**						
% incorp. residues early	43.3	32.8	28.0	25.0	16.7	6
% incorp. residues late	35.8	36.5	60.0	39.3	33.3	21
% burned residues	10.4	19.0	4.0	21.4	16.7	57.6
% removed residues	4.5	7.3	4.0	17.9	33.3	12.1


*MzSol, sole-cropped maize; MzLeg, maize intercropped with at least one legume; MzOth, maize intercropped with at least one non-legume; LegSol, sole-cropped legume; LegLeg, two or more legumes intercropped (no maize); includes one legume intercropped with a non-legume (no maize); Cash and Other, cotton or tobacco (no maize); includes any cropping system not falling into the above categories. Based on survey subset of farmers with detailed fertility management and area questions (*n* = 220) conducted July of 2013 in Central Malawi.*

### Residue Management

Farmers reported using diverse practices for residue management. For example, some farmers incorporated residues early (i.e., soon after harvesting the crop); others incorporated residues late (i.e., in the following season, during land preparation); and still others burned residues on the field; or removed the residue to use for fodder, building materials, etc. A large majority of farmers reported incorporating residues on at least some fields (78%), while 11% removed the residues for other purposes and 26% of farmers reported residue burning (**Table 6**). We note that farmers commented that residue burning was sometimes beyond their control, carried out by other members of the community as a rodent hunting or pest control activity, where fire is not always confined to the intended field.

**FIGURE 5:**
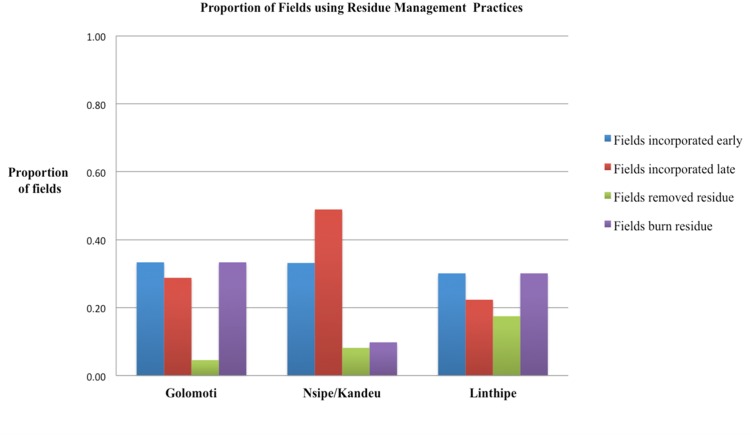
**The proportion of fields using a specific residue management practice presented in terms of low, medium, and high potential sites in Central Malawi.** Based on survey conducted July of 2013, field *n* = 600.

No clear relationship was observed for residue management across the sites in terms of an environmental gradient, as the largest number of farmers who practiced residue incorporation were in the medium potential sites, however, these farmers practiced contrasting patterns of incorporation. That is, the majority of farmers based in Kandeu reported practicing early residue incorporation (59%) whereas a modest number of farmers based in Nsipe (18%) practiced this, and a large majority (73%) in Nsipe opted for late residue incorporation during land preparation. Over one-third of farmers (38%) reported residue burning at both the marginal Golomoti site and the high potential-Linthipe site, whereas burning was minimal at the medium potential sites (14–16%). Nearly 16% of farmers in Kandeu and 23% of farmers in Linthipe reported crop residue removal, for various purposes. The patterns of residue management are shown in (**Figure 5**). Here we see that the proportion of fields with residue incorporation practices is common, compared to removal or burning of residues, which is practiced at all sites but particularly in the medium potential areas. Of those farmers who reported residue removal, some farmers used legume residues for fodder and soil improvement, while at a surprisingly number of farmers reported burning legume residues (21%). This compared to maize residues that were rarely removed (5%) or burned (10%). Residues from legume fields were incorporated early about 20% of the time; whereas in maize-based fields (including sole maize and maize-legume intercrops) residues were incorporated early about one-third of the time (**Table 7**).

## Discussion

The household socio-economic characteristics the 2013 survey suggests scarcity of resources for smallholder farmers across the four EPAs, and relatively large family sizes. The average dependency ratio of 108 observed in the study is higher than the overall average in Malawi which was reported in 2015 as 95 (World Bank, 2016). Farmers are cultivating small size land holdings averaging 0.85 hectares, which includes 1–2.4 fields. A modest diversity of crops were grown overall, that included a few dominant crops, notably maize, groundnut, tobacco that were grown regardless of the varied climatic factors (**Table 2**). Farmers also own few livestock relative to the region, but typical of Malawi thus suggesting that there is modest farm diversity in Malawi. This may be a result of the very high poverty, and limited market access. The rural population in Malawi is increasingly existing on small land parcels, which could be attributed to historical, cultural, and political socio-economic disparities. In 2010 GINI coefficient measure of 0.34 for Malawi suggests significant inequalities across the rural population (National Statistical Office of Malawi [NSO Malawi], 2013). This finding is also consistent with neighboring countries such as in Mozambique where rural farmer resource endowment is attributed to land ownership, livestock ownership, and historical events that impact the rural populations (Rusinamhodzi et al., 2016). We found significant challenges to promote increased agriculture production relying on intensification alone, as the resource base is, in many cases simply too degraded requiring multiple investments in input resources to support maintenance of production potential.

The characterization of maize-based systems from the Central Malawi action research sites of Africa RISING and nearby villages highlight farmer cropping system choices and investments being made in soil fertility. Generally, agricultural land was characterized as moderately to marginally suitable for production. Soil limits to productivity were compounded by a highly variable climate and topographically driven evapotranspiration that can limit yield potential of crop species not suited to high temperatures and poor water availability (**Table 1**). These biophysical limits to production interact, often unpredictably, with socio-economic elements to affect agricultural production. Classification of agricultural sites based on soil and environmental factors (**Figure 2**) such as soil properties, terrain, and precipitation analysis of these external influences to land suitability and gives us a general trend across scales for agricultural productivity in Malawi. Such agricultural land suitability maps could be used to inform agricultural extension workers and researchers across different land classification systems for particular regions and to assist the formulation of different approaches to address different sites and farmer needs.

Overall soil fertility status was marginal, particularly in the climatically risky lakeshore area of Golomoti. This is consistent with earlier findings regarding low soil organic matter and nitrogen availability countrywide on smallholder farms in Malawi (Snapp, 1998) and more broadly in the region (Tully et al., 2015). We found evidence of agricultural intensification practices, in terms of widespread fertilizer use across all sites, with 80% or more of farmers applying fertilizer to one or more fields. Golomoti had the lowest rate of fertilizer use, which may be related to high evapotranspiration and marginal soils at this site, which could increase the risk of unprofitable fertilizer application. Fertilizer use across the sites was in range of the Malawi government recommended N rate for maize (Chirwa et al., 2011).

Our findings suggest significant challenges to agriculture that relies on intensification alone, as the resource base is poor, with degraded soils and farmers have limited labor or land. Access to fertilizers has been enhanced in Malawi through subsidies, but in addition, investments in education and organic resources are required to support production potential being maintained in an increasingly marginal environment. Farmers across the sites were constrained in terms of resources to support use of organic amendments, including limited to nil livestock, and labor limitations (**Table 2**). Animal manure and compost applications was modest on most fields, presumably related to the limited manure resource availability but may have also been due to poor extension contact and labor constraints. Research has shown that Malawian farmers with livestock in the northern region use farmyard manure, and have benefited from incorporating manure into their systems. There appears to be less access to manure for soil fertility improvement in Central Malawi, due to a smaller animal population and greater density of farmers (Kabuli and Mar, 2006). Additionally, burning of residues is practiced; especially in the Dedza district were a third of farmers burn residues according to our findings. Past studies for Malawi show that farmers have a range of reasons for burning residues, including reduced labor requirements relative to land preparation without burning, and in some cases extension messages recommend burning to eradicate pests and weeds (Snapp S.S. et al., 2002). Burning and removal of crop residues reduces organic inputs, which can lead to loss of soil organic matter, an insufficient nitrogen (N) supply, ultimately decreasing soil water holding capacity and crop productivity. There is a considerable evidence base that complementary use of inorganic and organic fertilizer is essential for sustainable management of crop production in Southern Africa (Vanlauwe et al., 2011).

### Maize-Legume Technologies

Over 85% of farmers grew maize intercrops in the 2012–2013 growing season, with about three-quarters of those growing some combination of maize-legume intercrop (**Table 5**). This level of intercropping has been reported previously for the highly populated southern region of Malawi, but not for the central region (Shaxson and Tauer, 1992). Not all maize-legume intercrops have beneficial effects on resource sustainability, as some early maturing and poor nitrogen-fixing cultivars support high removal of nutrients in the form of harvested grain (Giller and Cadisch, 1995). However, the presence of highly vegetative, nitrogen-fixing legumes is an important sustainable practice, including many locally grown varieties of pigeonpea, cowpea, soybean, and mucuna such as reported here.

A previous Central Malawi survey conducted in the late 1990’s found a low proportion of land in legume crops (9–28%) and many barriers to legume production (Snapp S. et al., 2002). Another study in Northern Malawi also found very low proportion of land is allocated to legume crops on smallholder farms (Mhango et al., 2013). Our results indicate a much larger proportion of land in legume crops than that reported in previous studies, notably due to a large area devoted to maize-legume intercrops (**Figure 3**). It may be that in the intervening decade that input and output markets have improved, and provided support for legume production. For example, since 2011 the Malawi government has provided smallholder agricultural subsidies that included vouchers for seeds of modern varieties of groundnuts and common bean. However, other input factors may have acted as barriers, including a prioritization of maize seed and fertilizer vouchers, and the logistical challenges of government procurement of legume seed that is not available through formal seed markets and biologically is not conducive to rapid multiplication. This led to modest numbers of legume seed vouchers being distributed in most years (Dorward and Chirwa, 2011). The effectiveness of the fertilizer subsidy is reflected in the high levels of fertilizer use observed in this study, with 76–93% of farmers applying fertilizer. Farmer access to modest varieties of legumes crops doesn’t appear to have been effectively promoted in Central Malawi, and indeed there is evidence of declining legume crop area overall (Chibwana et al., 2012).

A surprising finding was that some farmers were growing combinations of two or more legumes in an intercrop system. This is an uncommon cropping system practice, in contrast to the combination of legume-cereal that is biologically complementary and a widespread practice on smallholder farms in Malawi and elsewhere (Shaxson and Tauer, 1992). Complementarity of legume crops can be achieved through the deliberate combination of early and late growth habits, with shallow versus deep rooting systems, to reduce competition for resources (Snapp and Silim, 2002). However, this doesn’t seem to be achieved by the farmer mixtures reported, which involved legumes with similar growth characteristics such as soybean and groundnut.

Maize and legume mixed production systems have the potential of increasing total soil C and N over time (Smith et al., 2016). Results from model simulations of soil C change over time are reported here for three of our sites, where continuous, unfertilized maize was associated with declines over time and maize in rotation with grain legumes maintained or saw modest accrual of soil organic C. For example, **Table 3** shows that Linthipe’s soil organic matter status is initially high, but is vulnerable to decline with removal of large amounts of grain compared to the other sites with lower yield potential. This is a somewhat surprising result indicating the value of crop and soil simulation models (Keating et al., 2003).

Also, legume presence in a maize intercrop has been shown to decrease soil surface exposure, for reduced soil moisture loss, and protection against soil erosion (Locke and Bryson, 1997). There have been reported productivity improvements through incorporating legume residues in other regions (Mureithi et al., 2005), although on-farm studies in Malawi have shown variable results (Snapp et al., 2010). Legume residue practices may be particularly beneficial for sites such as Golomoti that experience high evapotranspiration and Kandeu with intermittent dry conditions. The use of early residue incorporation in this study was highly variable within sites, by field and by crop. It was notably lacking for legume fields (**Table 7**). Further, almost a quarter of farmers burned legume residues, whereas only 10% of farmers burned maize residues. This is surprising given that legume residues have high nutrient content and are of value for livestock feed as well as soil building. This phenomena of legume residue burning has been observed previously in Malawi (Snapp S. et al., 2002).

Sustainable intensification requires integrated management that combines not only greater production of legumes, but also appropriate residue management and investment of complementary inputs such as fertilizer. As noted in a recent study, there is a paradox in that the poorest farmers may benefit the most from legume production, yet are rarely able to invest in inputs such as rhizobium and phosphorus, that support good legume agronomy (Franke et al., 2014).

Where extension support and other services are available to Malawi farmers (**Table 2**), as seen in the Linthipe and Nsipe areas, farmers may be able to access information on cropping techniques such as growing ‘best bet’ legume crops. These have been promoted in Malawi to contribute toward improving sustainability of intensive farming practices, by use of legume crops that combine highly vegetative growth habits with production of grain (Giller and Cadisch, 1995; Gilbert, 2004). However, there appears to have been almost no extension attention to residue management practices, and there is little knowledge of tradeoffs associated with early versus late season residue incorporation (Bezner Kerr et al., 2007).

### Policy and Community Partnership

In recent years, Malawi’s agricultural input subsidy program has increased access of improved inputs for many smallholder farmers by providing vouchers that enable access to agricultural inputs, primarily modern maize varieties and fertilizer (Dorward and Chirwa, 2011). However, Chirwa et al. (2011) found that farmers of the most marginal agricultural lands received few benefits. Further, there have been no complementary investments in extension education to support sustainable use of inputs, and farmer access to extension services in Malawi remains poor.

Increasing returns to rural agricultural investments in Malawi for resource poor farmers is a core goal of the Africa RISING program and related agricultural development initiatives.

Analysis that explores agricultural land suitability across scales, and modeling scenarios on using available agroecological and environmental datasets is advantageous to assist researchers and decision makers to monitor current agricultural practices and to identify knowledge gaps and complementary investments to support sustainable use of resources. We found evidence in this study of an increase in farmers applying fertilizer at recommended doses to maize production fields; compared to earlier studies. However, complementary investments in sustainable practices such as applying manure or investing in legume production remains widely neglected. Overall, farmer practices vary widely in terms of manure and crop residue management, and would benefit from extension advice.

## Conclusion

The results from this characterization paper highlight Malawi’s smallholder farmer’s current practices as constraints and opportunities for developing SI of agriculture. Marginalized farmers are at the center of most adoption strategies, working together with scientists and local extension agencies. SI progress for Malawi is challenged by many preconditions, for example; lack of local infrastructure, poor extension, and access to inputs, which is financially out of reach for most farmers (Sumberg, 2005). The study illustrates for a range of environmental conditions and local practice the implications for SI of agriculture. Crops grown and how soils and crops are managed, particularly crop residues, were found to vary markedly within and across districts. Fertilizer use was fairly widespread, yet variable, which may reflect the effect of not only the Malawi government’s investment in subsidies but the disconnect between access and knowledge with only a little over half of farmers receiving contact with extension services.

Sustainable intensification practices such as fertilizer application appear to be widespread, along with legume intercropping and growing maize, both hybrid and local varieties. However, management of organic inputs was inconsistent. Only 20–40% of farmers applied organic manure or compost to a field, and residue management of legumes includes some highly unsustainable practices such as burning and removal of residues from the field. Early residue incorporation is important for maximizing soil benefits from residues, and recycling of nutrients, however, it was primarily practiced on sole maize plots. This study can serve as a guide for a sustainable trajectory that emphasizes strengthening holistic agricultural development with decision makers and scientists working alongside marginalized farmers. Finally, SI practices for Southern Africa have great potential, yet further work is needed to support improved extension messages and consideration of the wide range of practices needed for sustainable, integrated crop management.

## Author Contributions

All authors made significant contributions to the conception and design of this paper, interpreting the data and in drafting the article, revising it critically for important intellectual content and gave final approval of the version to be submitted and any revised version in agreement to be accountable for all aspects of the work that relate to accuracy or integrity of any part of the work are appropriately investigated and resolved.

## Conflict of Interest Statement

The authors declare that the research was conducted in the absence of any commercial or financial relationships that could be construed as a potential conflict of interest.

## Footnotes

1 http://www.malawiproject.org/about-malawi/geography/ accessed 11 June 2016

2 ^2^(dataset source) Africa Soil Information Service (AFSIS) soil dataset, with terrain derived from SRTM DEM (90 m) (Li et al., submitted).
